# Limb Remote Ischemic Conditioning Ameliorates Cognitive Impairment in Rats with Chronic Cerebral Hypoperfusion by Regulating Glucose Transport

**DOI:** 10.14336/AD.2020.1125

**Published:** 2021-08-01

**Authors:** Changhong Ren, Yuanyuan Liu, Christopher Stone, Ning Li, Sijie Li, Haiyan Li, Zichao Cheng, Jiangnan Hu, Weiguang Li, Kunlin Jin, Xunming Ji, Yuchuan Ding

**Affiliations:** ^1^Beijing Key Laboratory of Hypoxia Translational Medicine, Xuanwu Hospital, Capital Medical University, Beijing, China.; ^2^Department of Endocrinology, The Affiliated Huai'an First People's Hospital of Nanjing Medical University, Huai'an, China.; ^3^Department of Rehabilitation Medicine, Affiliated 3201 Hospital of Xi'an Jiaotong University School of Medicine, Hanzhong, China.; ^4^Department of Neurosurgery, Wayne State University School of Medicine, Detroit, MI 48201, USA.; ^5^Beijing Institute of Brain Disorders, Capital Medical University, Beijing, China.; ^6^Department of Pharmaceutical Sciences, University of North Texas Health Science Center, Fort Worth, Texas 76107, USA.; ^7^Department of Pharmacology & Neuroscience, University of North Texas Health Science Center, Fort Worth, Texas 76107, USA.

**Keywords:** ovary, aging, pathophysiology, limb remote ischemic conditioning, cognitive impairment, glucose transport, adenosine monophosphate activated protein kinase, chronic cerebral hypoperfusion

## Abstract

Cognitive impairment is closely associated with the slowing of glucose metabolism in the brain. Glucose transport, a rate-limiting step of glucose metabolism, plays a key role in this phenomenon. Previous studies have reported that limb remote ischemic conditioning (LRIC) improves cognitive performance in rats with chronic cerebral hypoperfusion (CCH). Here, we determined whether LRIC could ameliorate cognitive impairment in rats with CCH by regulating glucose transport. A total of 170 male Sprague-Dawley rats were used. Animals subjected to permanent double carotid artery occlusion (2VO) were assigned to the control or LRIC treatment group. LRIC was applied beginning 3 days after the 2VO surgery. We found that LRIC can improve learning and memory; decrease the ratio of ADP/ATP; increase glucose content; upregulate the expression of pAMPKα, GLUT1 and GLUT3; and increase the number of GLUT1 and GLUT3 transporters in cerebral cortical neurons. The expression of GLUT1 and GLUT3 in the cortex displayed a strong correlation with learning and memory. Pearson correlation analysis showed that the levels of GLUT1 and GLUT3 are correlated with neurological function scores. All of these beneficial effects of LRIC were ablated by application of the AMPK inhibitor, dorsomorphin. In summary, LRIC ameliorated cognitive impairment in rats with CCH by regulating glucose transport via the AMPK/GLUT signaling pathway. We conclude that AMPK-mediated glucose transport plays a key role in LRIC. These data also suggest that supplemental activation of glucose transport after CCH may provide a clinically applicable intervention for improving cognitive impairment.

Vascular dementia (VaD) is type of major neurocognitive disorder caused by hypoxic or hemorrhagic brain tissue damage that accumulates over time due to various acute and chronic cerebrovascular diseases [1, 2]. Numerous studies have demonstrated that chronic cerebral hypoperfusion (CCH) is associated with the initiation and progression of both VaD and Alzheimer’s disease (AD). VaD is the second most common type of dementia after AD, accounting for 20% of all dementias; moreover, as society continues to age, this proportion is projected to triple by 2050 [3]. Unfortunately, despite this serious and increasing contribution to neurocognitive morbidity, the capacity to treat VaD is very limited. Beyond standard measures to control vascular risk factors such as hypertension and diabetes, only a small number of cholinesterase inhibitors have been clinically proven to be effective and even these only have modest improvement in cognitive function while failing to modify overall prognosis [4].

In this setting, there is an urgent need to discover and perfect new therapies designed to prevent the onset of VaD, ameliorate its progression, or both. One method that possesses potential to accomplish these goals is limb remote ischemic conditioning (LRIC). LRIC entails instituting a transient sublethal blood flow blockage of the distal limb that has been shown to induce endogenous protection and protect vital organs (such as the heart, brain, kidneys, etc.) from severe fatal ischemic injury [5]. As it gains recognition as an unconventional means to prevent and treat hypoxic-ischemic cerebrovascular diseases, LRIC has attracted the attention of an increasing number of research groups from all over the world. LRIC can effectively improve collateral circulation, increase cerebral blood flow in ischemic brain regions and reduce the recurrence rate of stroke [6-8]. In a recent study of relevance to VaD specifically, Wang et al. found that after one year of LRIC treatment, patients with cerebral small vessel disease and mild cognitive impairment had increased learning and memorization abilities along with significantly reduced volume of white matter degeneration as measured by brain MRI [9]. In a rodent model of focal cerebral ischemia, LRIC reduced infarct volume, promoted functional recovery after cerebral ischemia and improved cognitive functions [10-12]. Although it has been further reported that LRIC can improving learning and spatial memory in rodents and patients [7, 9, 13, 14], the exact mechanism of this improvement has not been fully elucidated.

Vascular diseases function in a multifaceted manner to cause chronic ischemia and hypoxia of the brain tissue, which in turn results in metabolic disorders [15], oxidative stress [16] and blood-brain barrier destruction [17, 18] among other deleterious changes including the induction of damage in neurons and white matter [14], all of which ultimately converge on the development of VaD. In the nervous system, the aerobic metabolism of glucose is the main source of energy in the form of adenosine triphosphate (ATP) [15, 19, 20]; consequently, any defects in or obstacles to glucose metabolism will precipitate a decline in cognitive function [21, 22]. The glucose transporter (GLUT) is especially important in this context, as it has been proven to be the rate-limiting step of glucose metabolism [19, 23]. In the mammalian brain, GLUT1 and GLUT3 are the predominant GLUTs responsible for glucose transport [24]. Mice with reduced GLUT1 levels display an age-dependent decrease in cerebral blood flow, glucose uptake and cognitive function [21]. Similarly, decreased GLUT1 and GLUT3 have been observed in the AD brain in humans and contribute to the impairment of brain glucose uptake/metabolism [25, 26]. Adenosine monophosphate activated protein kinase (AMPK) is involved in this process as a molecular center for the control of cell energy metabolism. It is found widely distributed in brain tissues and is responsible for controlling the level of energy metabolism in cells by regulating the expression of GLUT genes [27] and its accumulation was found to be associated with a protective effect conferred by remote ischemic conditioning against cerebral hemorrhage [28].

To date, despite the considerable plausibility generated by previous work, no direct evidence that LRIC promotes learning and memory associated with AMPK-mediated glucose uptake in CCH, has been shown. Consequently, the implications of this promising new therapy for the treatment of VaD have not been fully explored. In this study, we attempted to explore these implications by using a CCH rat model subjected to LRIC and evaluate the impact of LRIC on cognitive impairment through the regulation of glucose transport via the AMPK/GLUT signaling pathway.

## MATERIALS AND METHODS

### Animals

All animal experiments were approved by the Animal Care and Use Committee of Xuanwu Hospital, Capital Medical University, China and conducted according to National Institutes of Health guidelines. Adult male Sprague-Dawley rats (220 to 260 g in weight) were purchased from Vital River Laboratories, Beijing, China and maintained on a 12-hour light/dark cycle with unlimited access to food and water.

### CCH model

The CCH model was established using the double carotid artery occlusion (2VO) model, as described previously [29]. In brief, rats were anesthetized with 4% enflurane and maintained in an anesthetic state with a mixture of 70% N_2_O and 30% O_2_ containing 1.5-2% enflurane using a small animal anesthesia system. The anesthetized rats were fixed on the operating table in the supine position. After routine skin preparation, an incision was cut on the skin in the middle of the neck. The bilateral common carotid arteries were carefully separated from the cervical sympathetic and vagus nerves. One carotid artery was then ligated with a silk thread. After 15 minutes, the contralateral carotid artery was also ligated. Finally, the neck incision was sutured. During the surgery, rectal temperature was monitored and maintained at 37±0.5 °C with a heat blanket. Rats in the sham group underwent the same surgical procedure except that the carotid arteries were not ligated.

### LRIC treatment

LRIC was performed as previously described [8] with some modifications. LRIC treatment started on the third day after 2VO surgery and was administered once daily until tissue collection. First, rats were anesthetized with sodium pentobarbital (30 mg/kg) intraperitoneally. Self-developed rat LRIC instruments were then used (Patent No. ZL201720524103.7); rat limb cuffs were wrapped on each hind limb and LRIC was performed for three cycles at 10 mins/cycle at 240 mmHg with intervening 10-min reperfusion intervals. Rats in the sham and 2VO groups were anesthetized similarly but did not undergo LRIC treatment thereafter. LRIC is implemented once a day until the animal is euthanized.

**Figure 1.**
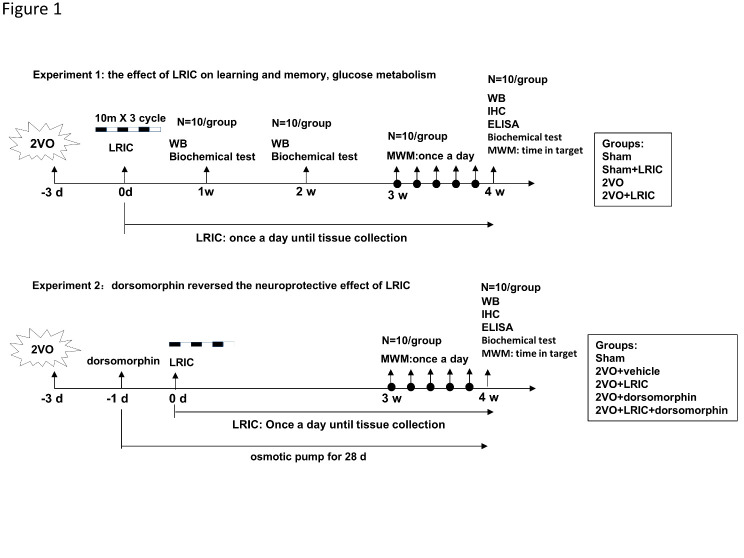
Experimental design and animal groups. 2VO, double carotid artery occlusion; WB, Western blot; ELISA, Enzyme-linked immunosorbent assay; IHC, Immunohistochemistry; MWM, Morris water maze; LRIC, Limb remote ischemic conditioning.

### Experimental design

Animals were divided into groups for two experimental studies in a randomized fashion using Excel-generated random numbers and experiments were performed in a blinded manner (Fig. 1). Experiment 1: To evaluate the effect of LRIC on learning and memory, and glucose metabolism, rats were randomly divided into the following groups: (i) sham, (ii) sham+LRIC, (iii) 2VO and (iv) 2VO+LRIC. Experiment 2: To explore the underlying mechanisms of LRIC on glucose transport, we administered dorsomorphin, a selective AMPK inhibitor. Rats were randomly assigned to the following groups: (i) sham, (ii) 2VO+vehicle, (iii) 2VO+LRIC, (iv) 2VO+dorsomorphin and (v) 2VO+LRIC+dorsomorphin.

### Morris water maze

The spatial learning and memory abilities of the rats were evaluated using the Morris water maze (MWM, DMS-2, Beijing, China) on the 21st day (i.e., after 3 weeks of LRIC treatment), as described previously [7]. The water maze used in our study was a flat, black, galvanized metal tank with a diameter of 150 cm and a height of 50 cm, within which a platform of 10 cm in diameter and 23 cm in height was located. The tank contained 25 cm of water and the platform was placed 1-2 cm below the surface of the water. The training maze was not visible. The maze was divided into four equal quadrants (Ⅰ, Ⅱ, Ⅲ, Ⅳ). In addition, a fixed visual cue (i.e., paper attached to the wall) was provided in the room. A camera was located above the center of the maze to record the movement of the rats and to send the data generated from this movement to an automatic tracking system (Noldus, EthoVision-XT 11).

The water maze experiment was completed within 6 days. For the first 5 days, the platform was fixed in the center of quadrant Ⅳ and rats were placed at different locations in the tank to train four times per day for 120-seconds sessions. If the rats did not find the platform within 120 s, they were picked up and placed on the platform for 20 seconds; if they did find it within 120 s, they were immediately removed from the platform. On the sixth day, an exploratory test was performed; the platform was removed, and the rats were allowed to swim and search for the platform for 60 s. All data were recorded using the EthoVision-XT 11 software, including the time spent to look for the target quadrant, the frequency with which rats crossed the position formerly occupied by the platform, the speed of swimming and the distance covered. The observer was blinded to the experimental conditions while performing the Morris water maze.

### Tissue collecting and processing

After 1, 2 and 4 weeks of LRIC treatment, rats were anesthetized by intraperitoneal administration with 400 mg/kg chloral hydrate and then perfused with saline. Left hemispheric tissues were taken, fixed in 4% PFA for 24 h and stored in sucrose solution (30% PBS) at 4 °C for 48 h. Then, brain tissues were embedded in a medium for frozen tissue specimens and stored at -80 °C for immunohistochemical staining. The right hemispheric cortex was immediately removed and placed on ice. Cortical tissues were then carefully separated and stored at -80 °C for biochemical, ELISA, and Western blot analyses.

### Biochemical testing

The ratio of ADP/ATP in the rat cortex homogenate was determined using a chemiluminescence kit (Sigma-Aldrich, Saint Louis, USA) according to the manufacturer’s protocol. The contents of glucose, lactic acid and pyruvic acid were determined using detection kits (Nanjing Jiancheng Biotechnology Co., Ltd, Nanjing, China) according to the manufacturer’s protocol as well. All assays were performed in triplicates. The observer was blinded to the experimental conditions.

**Figure 2.**
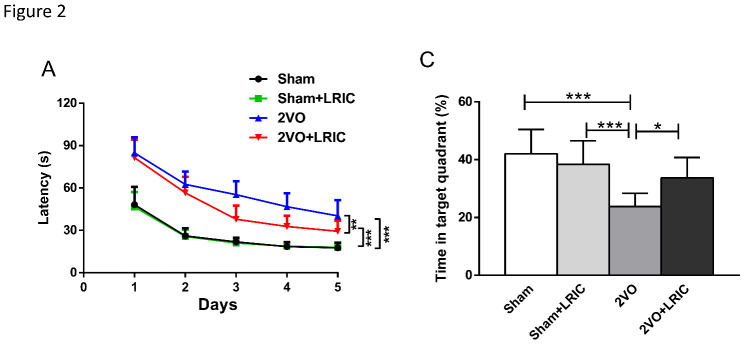
LRIC improved learning and memory in 2VO rats using the Morris water maze. (A) Escape latency time determined during Morris water maze testing beginning 3 weeks after LRIC treatment. (B) Percentage of time spent in the target quadrant. *P<0.05, **P<0.01, * **P<0.001, n=10/group.

### Western blot

The stored cerebral cortex tissues were weighed and then lysed in RIPA buffer using an ultrasonicator. The supernatant was extracted, and protein concentration was determined using the BCA method. A total of 60 μg of protein was separated using SDS-PAGE and transferred onto a PVDF membrane. The membrane was blocked at room temperature for 1 h and incubated overnight at 4 °C with primary antibodies against GLUT1, GLUT3 and pAMPKα (Cell Signaling, USA). The specific reaction was visualized using a chemiluminescent substrate (GE Healthcare, UK) and β-actin was used to verify equal loading (1:3000, Sigma-Aldrich, Missouri, USA). The optical density of protein was measured using *ImageJ software (NIH, Bethesda, MD, USA)*.

**Figure 3.**
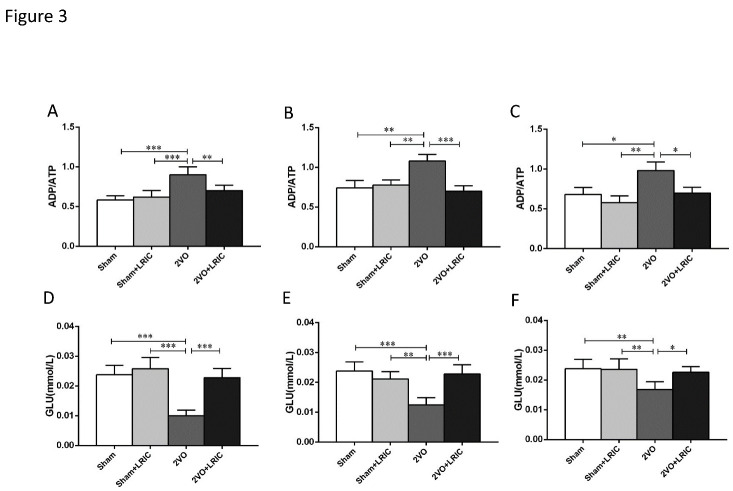
LRIC increased the content of ATP and glucose in the cortex of 2VO rats. (A and D) show the ratio of ADP/ATP and glucose content after 1 week of treatment. (B and E) show the ratio of ADP/ATP and glucose content after 2 weeks of treatment. (C and F) show the ratio of ADP/ATP and glucose content after 4 weeks of treatment. *P<0.05, **P<0.01, ***P<0.001. Bar graphs are mean±SD, n=10/group.

### Enzyme linked immunosorbent assay (ELISA)

The contents of GLUT1 and GLUT3 in the cortex were determined using ELISA kits (EK14283, EK14285, respectively, Signalway antibody). The cerebral cortex was homogenized in 5 mL of PBS with a glass homogenizer on ice. The resulting suspension was sonicated with an ultrasonic cell disrupter. Thereafter, the homogenates were centrifugated for 5 mins at 5000 × g. The supernatant was assayed according to the manufacturer’s protocol. The observer was blinded to the experimental conditions.

### Immunofluorescence staining

Frozen brain tissues were sliced into 10 μm sections and prepared for immunostaining as described previously [30]. The primary antibodies were rabbit anti-NeuN (1:300, Invitrogen, USA), mouse anti-GLUT1 and mouse anti-GLUT3 antibodies (1:400, Santa Cruz, USA). The secondary antibodies were Alexa Fluor 488-, 594- conjugated donkey anti-mouse or anti-rabbit, (1:200; Invitrogen, Grand Island, NY, USA). Slides were mounted using ProLong Gold antifade reagent with DAPI (Molecular Probes, USA) and observed with a Nikon Ti Eclipse Epi-Fl Illuminator (Nikon, Japan). The images were taken from six fields in the cerebral cortex. The observer was blinded to the experimental conditions.

### Intracerebroventricular injection with osmotic pump

Intracerebroventricular administration and embedding of the osmotic pump were performed as previously described [31, 32]. Briefly, rats were placed in a stereotaxic apparatus under 2.5% enflurane anesthesia. A 28-day Alzet osmotic pump (Alza Corporation, Palo Alto, CA, USA) was implanted in a subcutaneous pocket on the back of the rats. A catheter connected to the pump was inserted into the lateral ventricle at the following coordinates based on the location of the bregma: anteroposterior 1 mm, right lateral 1.5 mm and depth 3.5 mm. The AMPK-specific inhibitor, dorsomorphin (0.1 μmol/day, purity ≥ 98%, P5499, Sigma-Aldrich, St. Louis, MO, USA), was dissolved in 20% DMSO in PBS. The Alzet pumps then delivered either dorsomorphin or the vehicle at a rate of 1 μl/h over 28 days. Treatment was blinded to the operator and randomized before surgery by drawing lots. Drug administration was maintained during behavioral tests.

**Figure 4.**
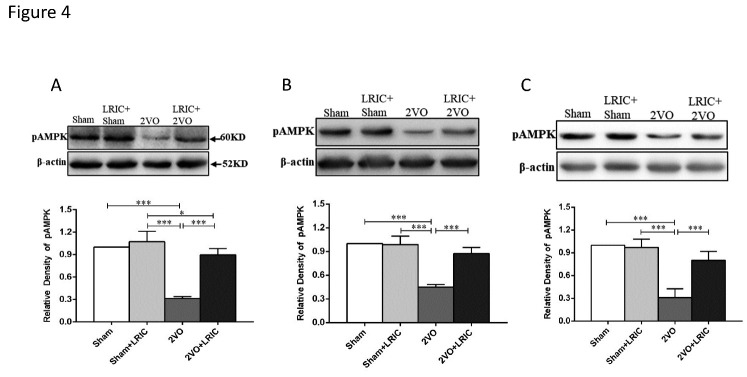
LRIC increased levels of pAMPK in the cerebral cortex as detected by Western blotting. (A and D) show levels of pAMPKα at 1 week after treatment initiation. (B and E) show levels of pAMPKα at 2 weeks after treatment initiation. (C and F) show levels of pAMPKα at 4 weeks after treatment initiation. *P<0.05, **P<0.01, ***P<0.001. Bar graphs are mean±SD, n=10/group.

### Statistical analysis

All data are expressed as mean ± standard deviation. Escape latencies were compared using repeated measures ANOVA. Differences in other parameters between different groups were analyzed using one-way ANOVA and Tukey’s multiple comparison test. These analyses were performed using SPSS 24.0 software. A value of P<0.05 was considered to be statistically significant.

## RESULTS

### LRIC improved learning and memory ability in 2VO rats

LRIC treatment was initiated on the third day after establishment of the 2VO model in rats. The Morris Water Maze was then used to test the effect of LRIC on cognitive impairment after CCH on the 21^st^ day, i.e., after 3 weeks of treatment. During the learning trials, the rats in all groups showed significant improvements in escape time latency (P<0.01, repeated-measures ANOVA). The mean escape latency over a period of 5 days for the 2VO group and the 2VO+LRIC group was significantly longer than the sham group, while the mean escape latency of 2VO+LRIC group was shorter than the 2VO group (P<0.05, repeated-measures ANOVA; Fig. 2A). The Morris water maze test on day 6 showed that the percentage of time spent in the target quadrant by the 2VO group was significantly lower than that of rats in the sham group (P<0.01, Fig. 2B) and that LRIC treatment significantly increased the time in the target quadrant compared with the rats in the 2VO group (P<0.05).

### LRIC increased ATP and glucose in the cortex of 2VO rats

The cerebral cortex is an area of vigorous energy metabolism and is responsible for cognitive function. Consequently, the effects of CCH are noted first in the associative cortical areas and spread diffusely thereafter via neuronal networks to impact the temporal and parietal cortices [33, 34]. With this pathogenic progression in mind, we selected the cerebral cortex as the region in which to record metabolic indices, such as glucose content and the ADP/ATP ratio. The ADP/ATP ratio in the cortex of 2VO rats was significantly higher than in the sham group while LRIC treatment for 1, 2 and 4 weeks was found to significantly reduce the ratio of ADP/ATP in the cortex of 2VO rats (P<0.01, P<0.001 and P<0.05, respectively, Fig. 3A-3C). Findings for glucose (GLU), which serves under normal physiological conditions as the main energy source for the brain, were similar [24]. As shown in Fig 2, GLU content in the cortex of 2VO rats was lower than that in the sham group while LRIC treatment for 1, 2 and 4 weeks significantly increased glucose content in 2VO rats (P<0.001, P<0.001 and P<0.05, respectively, Fig. 3D-F).

**Figure 5.**
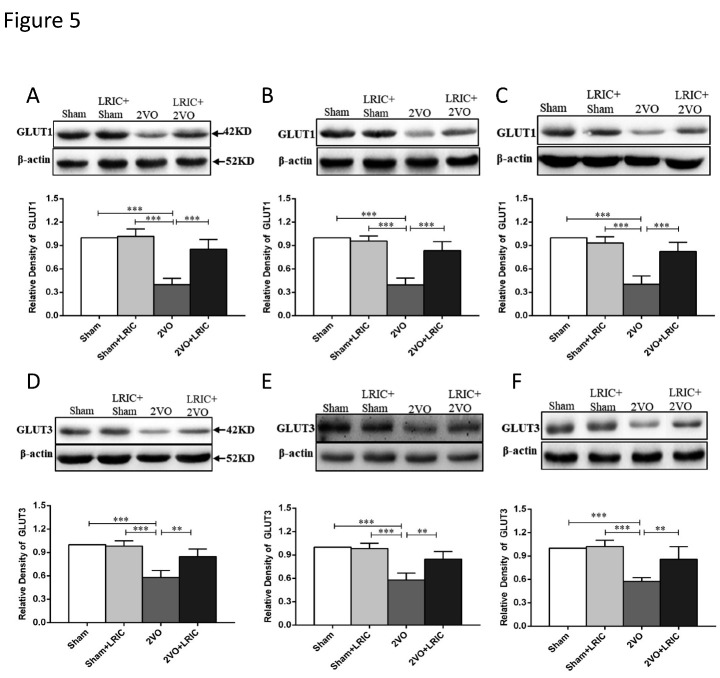
LRIC increased levels of GLUT1 and GLUT3 in the cerebral cortex as detected by Western blotting. (A and D) show levels of GLUT1 and GLUT3 at 1 week after treatment. (B and E) show levels of GLUT1 and GLUT3 at 2 weeks after treatment. (C and F) show levels of GLUT1 and GLUT3 at 4 weeks after treatment. *P<0.05, **P<0.01, ***P<0.001. Bar graphs are mean±SD, n=10/group.

### LRIC increased levels of glucose transport-related proteins pAMPKα, GLUT1 and GLUT3 in the cortex of 2VO rats

**Figure 6.**
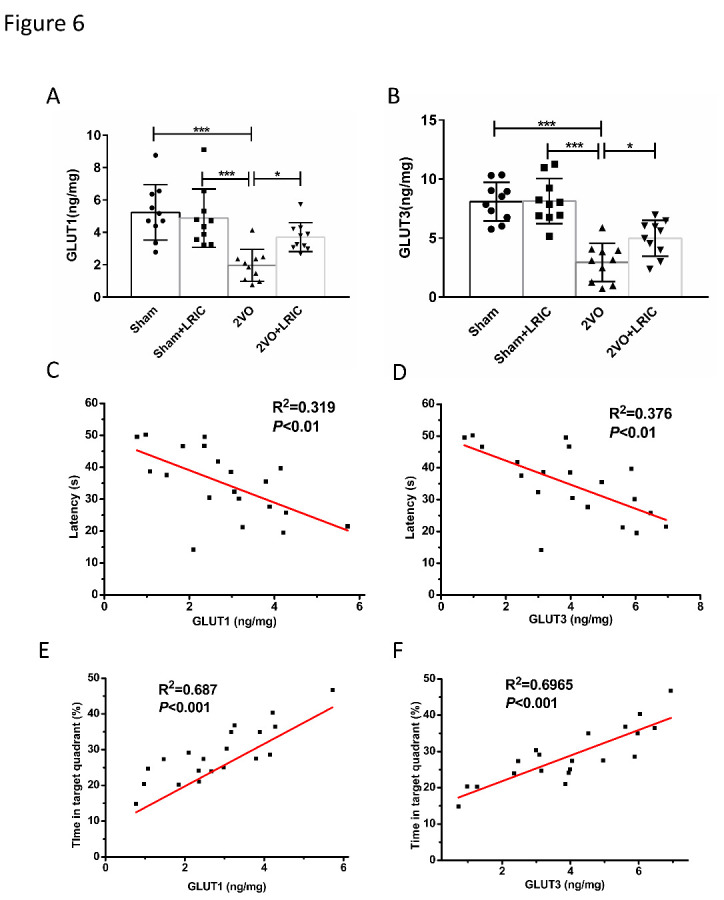
LRIC increases levels of GLUT1 and GLUT3 in the cerebral cortex as detected by ELISA. (A and B) show the data obtained by ELISA in rats at 4 weeks after treatment. *P<0.05, ***P<0.001. Bar graphs are mean±SD, n=10/group. (C and D) show the Pearson correlation between escape latency time on the fifth day of training and GLUT1 and GLUT3, respectively. (E and F) show the Pearson correlation between time in the target quadrant on the fifth day of training and GLUT1 and GLUT3, respectively.

As assessed by Western blotting, levels of pAMPKα were significantly decreased in the cortex of 2VO rats and significantly increased after 1, 2 and 4 weeks of LRIC treatment (P<0.001, P<0.001 and P<0.001, respectively; Fig. 4A-C). We also measured the expression levels of GLUT1 and GLUT3 in the cortex using Western blot, finding that, when compared with the sham group, levels of GLUT1 and GLUT3 in the cortex of 2VO rats were significantly decreased. LRIC treatment for 1, 2 and 4 weeks increased levels of GLUT1 and GLUT3 compared with those found in the 2VO group (P<0.001 for all, Fig. 5D-F). We further quantified the content of GLUT1 and GLUT3 in the cortex by ELISA at 4 weeks after LRIC treatment. Consistent with the Western blot results, LRIC significantly increased GLUT1 and GLUT3 levels as assessed by ELISA (Fig. 6A and B).

To investigate whether the increased levels of GLUT1 and GLUT3 obtained with LRIC treatment correlated with cognitive improvement, Pearson product linear regression analysis was performed. Results demonstrated that, after 4 weeks of LRIC treatment, the content of GLUT1 and GLUT3 in the cortex were negatively correlated with latency time (Figure 6C and D) and positively correlated with the percentage of time spent in the target quadrant during the Morris Water Maze test (Fig. 6E and F). These results suggest that upregulation of GLUT1/GLUT3 may be an important component of LRIC-induced cognitive improvement in the context of CCH.

### LRIC significantly increased expression of GLUT1 and GLUT3 in the cortical neurons of 2VO rats

**Figure 7.**
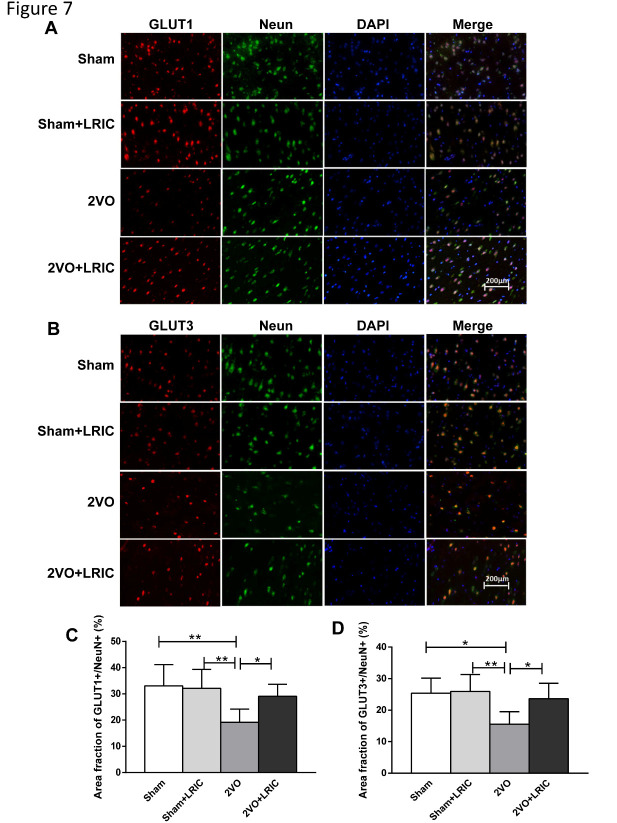
Immunofluorescence revealed that LRIC increased expression levels of GLUT1 and GLUT3 in the cortical neurons of 2VO rats. (A and B) show representations of immunofluorescence co-staining for GLUT1, GLUT3 and NeuN in the cerebral cortex. Scale bar=200 μm. (C) Bar graphs show the areas of GLUT1^+^/NeuN^+^. D. Bar graphs show the areas of GLUT3^+^/NeuN^+^. **P<0.01, ***P<0.001. Data shown are mean±SD, n=10/group.

Neurons in the rat cortex were co-stained with GLUT1 or GLUT3 at 4 weeks after LRIC treatment initiation. GLUT1- or GLUT3-positive cells were stained red, NeuN-positive cells were stained green and GLUT1- or GLUT3- and NeuN-positive cells are shown in yellow (Fig. 7A and B). Compared with the sham group, expression levels of GLUT1 and GLUT3 in the cortical neurons of 2VO rats were decreased. In the LRIC group, expression levels of GLUT1 and GLUT3 in the cortical neurons of 2VO rats increased (P<0.05 and P<0.05, respectively; Fig. 7C and D).

### Dorsomorphin abolished the beneficial effects of LRIC on learning and memory in 2VO rats

**Figure 8.**
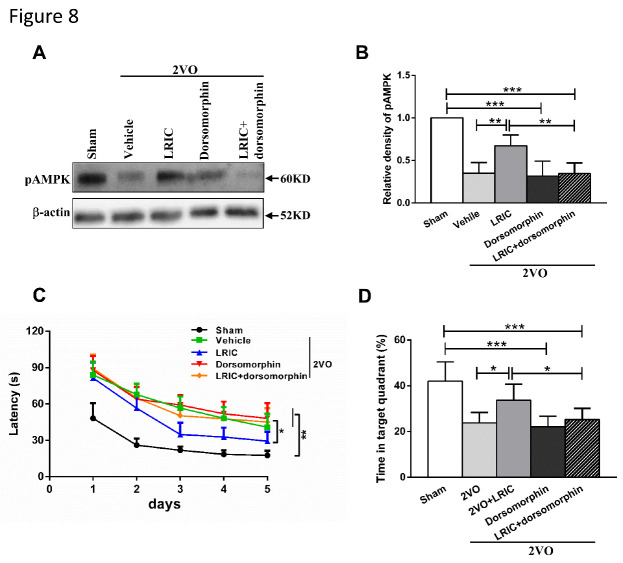
Dorsomorphin abolished the beneficial effects of LRIC on learning and memory in 2VO rats. Intracerebroventricular administration of dorsomorphin decreased the expression of pAMPKα in the cortex at 4 weeks after treatment. (A) Representative Western blot bands. (B) Quantitative analyses of pAMPKα expression. **P<0.01, ***P<0.001. Data shown are mean±SD, n=7/group. (C) Escape latency time during the Morris water maze test after 3 weeks of LRIC treatment. (D) Percentage of time spent in the target quadrant. *P<0.05, **P<0.01; ***P<0.01, n=10/group.

First, we found by Western blotting that inhibition of AMPK with dorsomorphin significantly decreased the expression of pAMPKα in both 2VO and LRIC-treated rats (Fig. 8A and 8B). LRIC without dorsomorphin improved the learning and memory ability of 2VO rats, but this effect was reversed with the administration of dorsomorphin (Fig. 8C and D); escape latency times were significantly increased in the LRIC+dorsomorphin group compared with the LRIC group (P<0.05). Correspondingly, the percentage of time spent in the target quadrant was significantly decreased in the LRIC+dorsomorphin group compared with the LRIC group (P<0.05).

### Dorsomorphin abolished the effects of LRIC on glucose and ATP contents and on GLUT1 and GLUT3 expression levels

**Figure 9.**
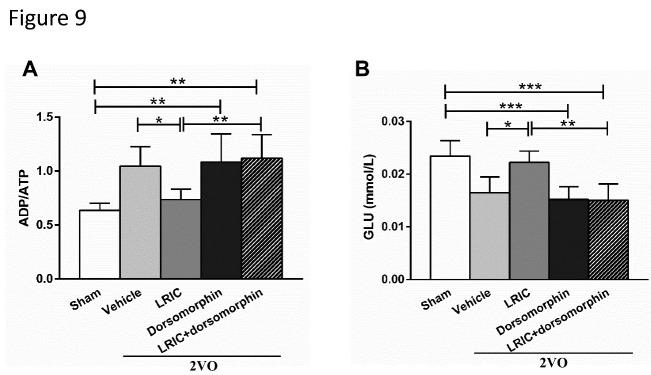
Dorsomorphin abolished the effect of LRIC on glucose and ATP contents. (A) This bar graph shows the ratio of ADP/ATP at 4 weeks after treatment initiation. (B) This bar graph shows glucose content at 4 weeks after treatment initiation. *P<0.05, **P<0.01, * **P<0.001, n=7/group.

**Figure 10.**
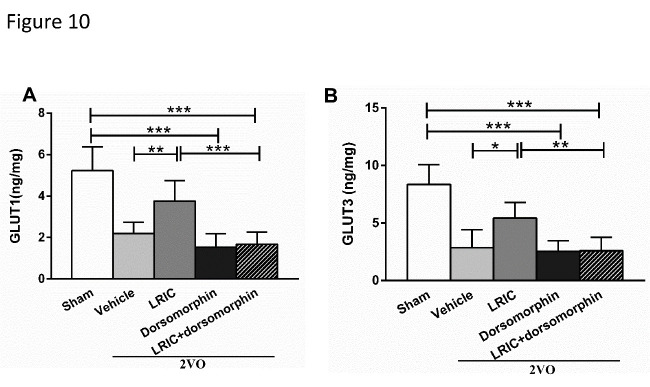
Dorsomorphin abolished the effect of LRIC on the expression of GLUT1 and GLUT3 detected by ELISA. (A) This bar graph shows the expression of GLUT1 after 4 weeks of treatment. (B) This bar graph shows the expression of GLUT3 after 4 weeks of treatment. *P<0.05, **P<0.01, ***P<0.001, n=7/group.

To explore whether dorsomorphin could inhibit the effects of LRIC on glucose and ATP contents, we measured glucose and ATP contents in the cortex after 4 weeks of LRIC treatment. Inhibition of AMPK with dorsomorphin significantly decreased both glucose and ATP levels (Fig. 9A and B). We also quantified the expression levels of GLUT1 and GLUT3 in the cortex using ELISA after 4 weeks of LRIC treatment. Inhibition of AMPK with dorsomorphin significantly decreased the expression of GLUT1 and GLUT3 (Fig. 10A and 10B).

## DISCUSSION

Here, we demonstrated that LRIC improved learning and memory after institution of a rat model of CCH. We made the following novel observations: (1) LRIC effectively increased glucose and ATP levels in the CCH rat; (2) The improved glucose metabolism secondary to LRIC treatment was mediated in part through increased expression of pAMPKα, GLUT1 and GLUT3; (3) Importantly, the expression levels of GLUT1 and GLUT3 in the cortex displayed a strong correlation with learning and memory as assessed using the Morris Water Maze test; (4) Treatment with dorsomorphin reversed the beneficial effects of LRIC on learning and memory, glucose and ATP contents and expression levels of GLUT1 and GLUT3. Taken together, these findings suggest that LRIC improved learning and memory in CCH rats via improvements in AMPK-mediated glucose uptake.

Cognitive abilities, such as learning and memory, are closely linked with the level of glucose metabolism in the brain [35]. Different brain regions have different glucose consumption levels, with the cerebral cortex, medial and lateral geniculate bodies and thalamus exhibiting particularly robust rates [36]. Therefore, we chose the cerebral cortex as the location at which to evaluate the energy metabolism status of 2VO rats by measuring glucose content and found that the glucose content in the cortex of these rats decreased significantly. Since glucose is ultimately metabolized to produce ATP, which supplies energy for various brain activities [19], we also measured ATP content in rat cortical areas. As expected, the ATP content in the cortex of 2VO rats was also significantly reduced (i.e., exhibited an increased ADP/ATP ratio).

In recent years, LRIC has garnered increasing recognition as a novel treatment for cerebrovascular diseases, yielding protective effects against acute ischemic stroke [37], symptomatic intracranial atherosclerosis [6] and chronic cerebral ischemia [7, 14]. Despite these encouraging clinical results, however, the responsible mechanism remains unclear. A number of studies have attempted to investigate this mechanism, finding that LRIC can reduce the production of free radicals [38], promote cell survival pathways [39] and regulate the immune system [40]. Our study adds another contribution to this list by demonstrating that LRIC significantly increases glucose metabolism in the cortex of 2VO rats; compared with the 2VO group, the glucose and ATP content in the cortex of rats in the LRIC+2VO group were significantly increased, while levels of lactic acid and pyruvate were significantly decreased. These results suggest that LRIC enhances cortical energy metabolism in 2VO rats by increasing the level of oxidative phosphorylation of glucose.

Maintenance of metabolic activity in mature brain tissue relies almost exclusively on the continuous oxidation of glucose [41]. Glucose is transported into the brain through the blood-brain barrier system by glucose transporters (GLUT) [42]. So far, 13 subtypes of GLUT have been found in mammals, of which GLUT1 and GLUT3 are widely expressed in the brain and considered the main glucose transporters in brain tissue [43]. Landau et al. reported that GLUT1 and GLUT3 expression was downregulated in the brain tissue of patients with Alzheimer's disease and glucose uptake was reduced, resulting in decreased cognitive function [44]. This link between glucose uptake and cognitive impairment has been linked to vascular insufficiency in recent studies showing that mice with reduced GLUT1 levels exhibit cognitive dysfunction associated with decreased brain capillary density, cerebral blood flow and glucose uptake, as well as increased blood-brain barrier leakage [21]. Similarly, studies utilizing knockout mice have confirmed that GLUT1^+/-^ mice have reduced brain volume and abnormal motor behavior, while GLUT3^+/-^ mice exhibit abnormal spatial learning and working memory [45, 46]. In this study, immunofluorescence staining of the cortical neurons of 2VO rats for GLUT1 and GLUT3 revealed a significant decrease in the expression levels of these glucose transporters. Moreover, as the expression levels of GLUT1 and GLUT3 in the cortex displayed a strong correlation with learning and memory as assessed by the Morris water maze test, our findings suggest that GLUT1- and GLUT3-regulated glucose metabolism in the cerebral cortex can play a key role in the cognitive function of 2VO rats.

Any factor that increases the ratio of AMP/ADP (such as starvation, oxidative stress, or glucose deprivation), can activate AMPK, which is responsible for the regulation of energy balance by acting on a number of downstream target proteins in multiple signaling pathways [47]. Specifically, studies have shown that AMPK serves as the molecular backbone of cellular energy metabolism, regulating intracellular energy processing by phosphorylation of metabolic enzymes and determination of GLUT gene expression throughout the brain [27]. It is also involved in the adaptation to cerebral ischemia and hypoxia; when these insults occur, increased expression of activated AMPK rapidly activates the glycolysis pathway in astrocytes to generate energy for neurons [48]. Our study found that pAMPKα levels were decreased in the cortical neurons of 2VO rats, but that LRIC significantly increased pAMPKα levels. Meanwhile, LRIC also significantly increased the expression levels of GLUT1 and GLUT3 in cortical neurons. These trends of change in pAMPKα, GLUT1 and GLUT3 levels are consistent with those of glucose and ATP. Therefore, we hypothesized that pAMPKα may be an important regulator of cortical glucose metabolism in 2VO rats by augmenting GLUT1 and GLUT3 expression. To confirm this mechanistic link, a specific inhibitor of AMPK was administered; this intervention exerted a number of deleterious effects, including reversal of the neuroprotective effects of LRIC, as shown by increased escape latency time and reduced time in the target quadrant during the MWM test; increase in the cortical ratio of ADP/ATP and reduction of glucose content; and reduction of GLUT1 and GLUT3 expression levels.

In conclusion, the results of this study indicate that LRIC can increase the expression levels of GLUT1 and GLUT3 and increase glucose metabolism in the cerebral cortex by activating pAMPKα, thereby improving the spatial learning and memory abilities of CCH rats. LRIC therefore appears to be a viable treatment to effectively improve cognitive function in CCH. Given its safety, non-invasiveness and low cost, LRIC constitutes a promising potential treatment for patients with VaD and other cerebrovascular diseases entailing cognitive impairment. In the future, more research should be conducted to further evaluate the efficacy of this treatment to pave the way toward its application in VaD patients, for whom current treatments are very limited. On the other hand, these data also suggest that supplemental activation of glucose transport after CCH may provide a clinically applicable intervention for the promotion of cognitive impairment.
